# Decreased expression of CYP27B1 correlates with the increased aggressiveness of ovarian carcinomas

**DOI:** 10.3892/or.2014.3666

**Published:** 2014-12-11

**Authors:** ANNA A. BROŻYNA, WOJCIECH JÓŹWICKI, CEZARY JOCHYMSKI, ANDRZEJ T. SLOMINSKI

**Affiliations:** 1 Department of Tumor Pathology and Pathomorphology, Oncology Centre, Prof. Franciszek Łukaszczyk Memorial Hospital, The Ludwik Rydygier Collegium Medicum, Nicolaus Copernicus University, 85-796 Bydgoszcz, Poland; 2 Department of Pathology and Laboratory Medicine, Division of Rheumatology, University of Tennessee Health Science Center, Memphis, TN 38163, USA; 3 Department of Medicine, Division of Rheumatology, University of Tennessee Health Science Center, Memphis, TN 38163, USA

**Keywords:** CYP27B1, ovarian cancers, prognostic factors, vitamin D

## Abstract

CYP27B1 hydroxylates 25-hydroxyvitamin D_3_ in position C1α into biologically active 1,25-dihydroxyvitamin D_3_, calcitriol. CYP27B1 is expressed in normal tissues and tumors. Since calcitriol indicates anticancer activities and CYP27B1 expression can be deregulated during malignant progression, we analyzed its expression in ovarian cancers in relation to pathomorphological features of tumors and overall survival (OS). Expression of CYP27B1 was evaluated in 61 ovarian tumors, 18 metastases and 10 normal ovaries. Normal ovarian epithelium showed the highest levels CYP27B1 with a significant decrease in its expression in ovarian cancers. Both poorly differentiated primary tumors and metastases showed the lowest level of CYP27B1 expression, while non-metastasizing tumors showed a higher CYP27B1 level than tumors that developed metastases. The expression of CYP27B1 was positively correlated with a lower proliferation rate, lower dynamism of tumor growth and tumor infiltrating lymphocyte response. Furthermore, CYP27B1 expression was negatively correlated with tumor cell modeling of their microenvironment. CYP27B1 expression was also associated with longer OS time. In summary, our results suggest that local expression of CYP27B1 in ovarian tumor cells can modify their behavior and promote a less aggressive phenotype by affecting local concentrations of active of vitamin D levels within the tumor microenvironment.

## Introduction

The active form of vitamin D_3_ is calcitriol [1,25-dihydroxyvitamin D_3_ (1,25(OH)_2_D_3_)], and shows pleiotropic activity in the human body (1–4). The classic role of calcitriol includes regulation of calcium and phosphate homeostasis in the body, while non-classic actions of vitamin D_3_ include regulation of other cellular and tissue functions. Calcitriol affects immunity (5,6), downregulates proliferation, and upregulates differentiation and apoptosis in normal and malignant cells of different origin (7–9). It also protects DNA against oxidative damage (10,11). Both calcitriol and other active forms of vitamin D_3_ show tumorostatic and anticarcinogenic effects against precancerous and cancerous lesions (12–17).

Vitamin D_3_ executes its biological functions through interaction with a vitamin D receptor (VDR), a member of the family of steroid hormone-activated nuclear receptors (18–20). After binding 1,25(OH)_2_D_3_, VDR heterodimerizes with retinoic acid X receptor (RXR) and after translocation to the nucleus acts as a transcription factor for genes with vitamin D response elements (VDREs). VDR has been found in almost all tissues of the human body (2,3). VDR was also found in tumor cells; however, its expression in these lesions is often decreased (21–25). Moreover, a VDR polymorphism is associated with increased risk of developing different cancer types, including melanoma, breast and lung cancers (26–28). Likewise, a polymorphism of the VDR gene is associated with a significantly higher susceptibility to ovarian cancer (29–31).

The biological active form of vitamin D_3_ is synthesized via a two-step hydroxylation. The first step of hydroxylation in the liver is catalyzed by CYP27A1 or CYP2R1, members of the cytochrome p450 family. The second reaction is catalyzed by 25-hydroxyvitamin D_3_ 1-α-hydroxylase (CYP27B1) in a distal convoluted tubule, the cortical and medullary part of the collecting ducts and the papillary epithelia of kidneys (32). However, the CYP27B1 enzyme is expressed ubiquitously in the body being found in several normal tissues such as skin, lymph nodes, colon, placenta, brain, breast placenta and adrenals (3,4,13,33,34). CYP27B1 is also expressed in malignant tissues including lung (35), colon (36) and breast cancers (37), and dysgerminomas (38). It is also expressed in immune cells (5). The extrarenal expression of CYP27B1 can increase the local level of biologically active calcitriol to regulate cellular and tissue functions at peripheral levels. Under physiological conditions, the CYP27B1 levels are regulated by calcitriol and VDR through a negative feedback mechanism (39). However, in pathological states this regulation can be disturbed or activation of cholecalciferol can be inhibited, resulting in decreased local levels of anticancerogenic 1,25(OH)_2_D_3_.

Since the 1990’s the role of vitamin D_3_ in the etiology and progression of several cancers has been well documented. Initial reports revealed the association between low serum levels of vitamin D and colon (40), prostate (41) and breast (42) cancers. A decreased vitamin D_3_ level is not only associated with a higher risk of cancer development but supplementation with calcitriol is also considered as a chemopreventive approach (43), which can also be used as an adjuvant during cancer treatment (10,44). Most recently, decreased serum levels of vitamin D were found in ovarian cancer patients (45). Moreover, ovarian cancer patients with severe deficiency of 25(OH)D_3_ (<10 ng/ml) showed a significantly reduced 5-year overall survival (OS). Another study revealed that vitamin D supplementation decreased ovarian cancer risk in postmenopausal women (46).

Ovarian cancer is the most common fatal cancer of the female reproductive system in industrialized countries. In Poland and in other European countries and in the USA, ovarian cancers are one of the most frequent genital tract malignancy in women with the highest mortality rate of all female cancers (47). The etiology and prognostic factors of ovarian cancers are still being investigated. Despite the great progress in the treatment of ovarian cancer, the 5-year OS is ~30–40%. Thus, better understanding of the molecular basis of ovarian cancer biology would help to optimize treatment procedures and/or develop possible new therapies. Since our previous study demonstrated decreased CYP27B1 expression during melanoma progression and its inverse correlation with Ki67 expression and OS time (48), we decided to analyze its expression in ovarian cancers in correlation with pathomorphological features.

## Materials and methods

### Patients

The first step of patient qualification was based on the clinicopathomorphological features from a digitized patient database of the Oncology Center in Bydgoszcz, Poland between 2006 and 2010. During this step, 74 ovarian tumor patients who underwent optimal cytoreductive surgery were selected. Of those, 13 cases were excluded due to lack of tumor presence in the blocks. Finally, the expression of CYP27B1 was analyzed in 88 formalin-fixed paraffin-embedded sections obtained from 66 patients, including 61 ovarian tumors, 18 metastases and 10 normal ovaries. The characteristics of the patients included in the present study are presented in Table I.

### Immunohistochemistry

The expression of CYP27B1 in ovarian tissues was detected using immunohistochemistry, as previously described (48). Briefly, formalin-fixed paraffin-embedded 4- to 5-μm sections were labeled overnight at 4°C with rabbit anti-CYP27B1 antibody (Santa Cruz Biotechnology, Santa Cruz, CA, USA) at a dilution of 1:75. The antigen-antibody binding was visualized with HRP-labeled anti-rabbit antibody and 3,3′-diaminobenzidine (DAB) (Envision System-HRP Labeled Polymer Anti-Mouse; Dako, Glostrup, Denmark), followed by hematoxylin counterstaining. The kidney sections served as positive control.

Furthermore, Ki67 immunostaining was performed as described previously (21,49,50).

### CYP27B1 immunohistochemistry assessments

Immunohistochemical evaluation of CYP27B1 was performed in a blinded manner without knowledge of the detailed histopathological diagnoses and other clinical data.

CYP27B1 staining was analyzed semiquantitatively. Both percentage and immunostaining intensity of CYP27B1 were evaluated. The staining intensity was assessed in relation to staining in kidney tissue using the scale from 0 to 3 arbitrary units (A.U.) with 0 as negative; 1, weak; 2, moderate; and 2, strong. The semiquantitative score (SQ-score) was calculated as follows: SQ = mean (IR × SI)/100, where IR is the percentage of immunopositive cells and SI is the staining intensity. Patients were stratified according to CYP27B1 SQ-scores as follows: SQ 0.0–0.99 = no CYP27B1, SQ 1.0–1.99 = low CYP27B1, and SQ 2.0–3.0 = medium CYP27B1. The SQ-score was evaluated within the central and border parts of the tumors.

### Evaluation of pathomorphological features

The categorization of the central (older) and border (new) parts of the tumors was carried out as previously described (50). Briefly, the former were the younger compartments of the malignant lesions with newly invading cancer cells and with signs of dynamic growth, while the latter were the older parts of the tumor without signs of dynamic growth.

Tumor-infiltrating lymphocyte (TIL) assessment followed similar analyses performed by others (51,52). Briefly, the following scale was used to asses lymphocytic infiltrate: 0, lack of lymphocytes; 1, single lymphocytic cells observed in one high-power field (HPF); 2, several lymphocytes (but <12) noted in one HPF; 3, a few dozen lymphocytes (but <100) noted in one HPF; 4, several hundred lymphocytes in one HPF; and 5, the number of lymphocytes was so high that it was incalculable.

**Figure 1:**
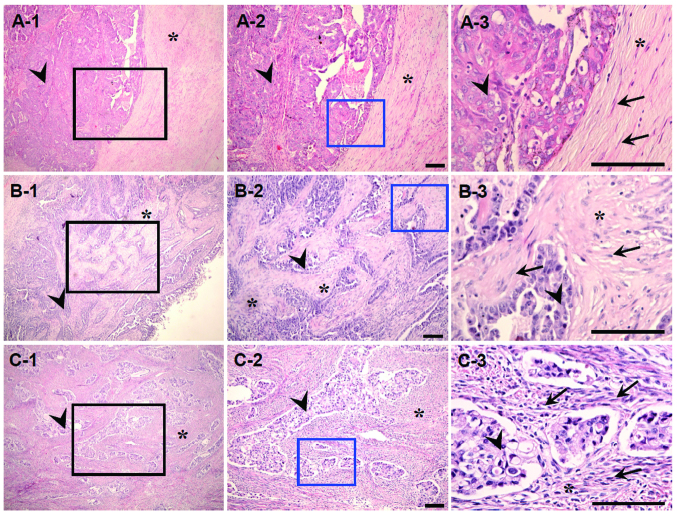
Ovarian cancer without modeling (A1–3) and with indistinct (B1–3) and distinct (C1–3) modeling (H&E staining). A-2, B-2 and C-2 present fragments indicated by black squares; A-3, B-3, C-3 present fragments indicated by blue squares. Asterisks indicate stroma, arrow heads indicate tumor nests, arrows indicate fibroblasts. Scale bars, 100 μm.

The stromal modeling pattern of cancer was defined as previously described (50). Briefly, an indication of dynamic dialogue between the tumor and the microenvironment was represented by an increased number of fibroblasts demonstrating dynamic mutual arrangements with cancer nests along with an increased number of inflammatory cells (e.g., macrophages). The modeling was graded as follows: lack, when no signs of such cross-communication were observed; indistinct, when only slightly visible; and distinct, when the dynamic cross-communication was observed. The ovarian cancer cases without modeling, with indistinct and distinct modeling are shown in Fig. 1A–C.

The tumor grade was evaluated in the whole tumor, as well as in sections immunostained with the anti-CYP27B1 antibody. Moreover, within the tumor section, CYP27B1 was evaluated separately in cancer compartments of different grades.

### Statistical analysis

Statistical analysis was performed with Prism 5.00 (GraphPad Software, San Diego, CA, USA). Results were considered to indicate a statistically significant result at p<0.05. Data are presented as means ± SD. For comparison of 2 or more groups, the t-test or one-way ANOVA were used. The Pearson’s correlation was used for evaluation of associations between immunostaining and categorical variables. Survival was calculated using the Kaplan-Meier method.

## Results

### CYP27B1 expression in relation to tumor grade and metastases

The expression of CYP27B1 was observed in 49 (80.3%) cases of primary ovarian tumors. Of those, 28 (45.9%) cancers showed low and 21 (34.4%) showed medium CYP27B1 levels. No single case with high CYP27B1 immunostaining was noted. Within metastases, 1 (5.6%) case showed no CYP27B1 immunostaining; 13 (72.2%) showed low; and 4 (22.2%) showed medium CYP27B1 immunostaining. Thirteen cases of matched pairs of primary cancer and metastases were analyzed. In 3 pairs, there were no changes in CYP27B1 expression in metastases vs. the primary lesions, in 5 cases a lower CYP27B1 level was observed in the metastatic tumors, and in 5 cases lower CYP27B1 immunostaining was recorded in the primary cancers.

**Figure 2:**
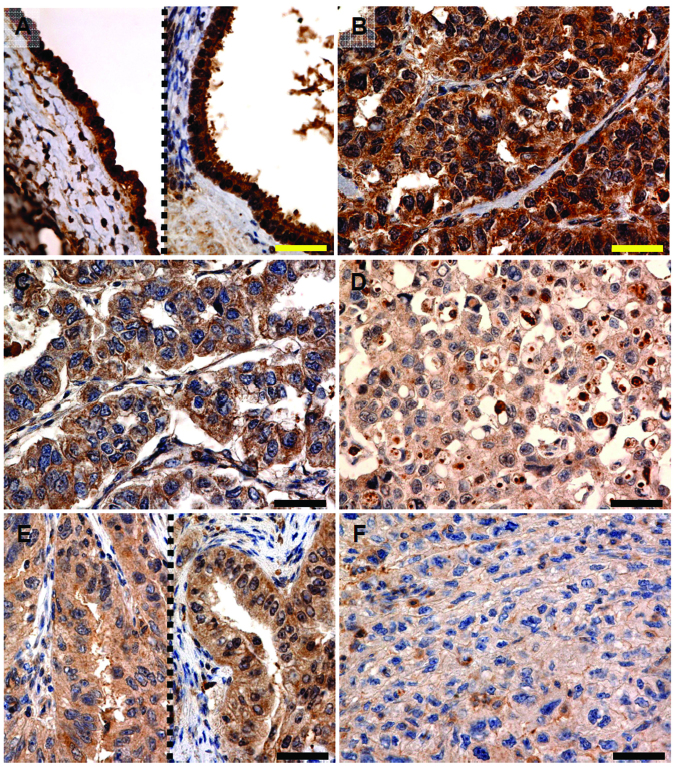
Representative CYP27B1 immunostaining in normal epithelium (of surface and follicle) (A), primary adenocarcinoma of grade G1 (B), G2 (C) and G3 (D) and metastases of grade G2 (E) and G3 (F). Scale bars, 50 μm.

**Figure 3:**
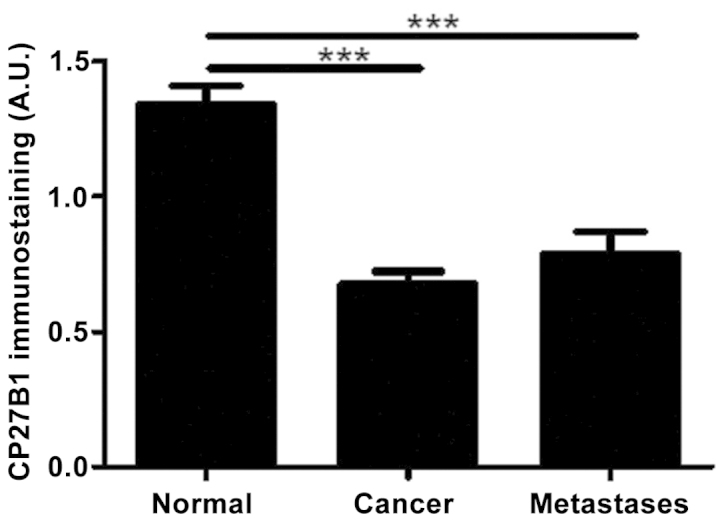
CYP27B1 immunostaining is inversely associated with cancer progression. Statistically significant differences are denoted with asterisks by ANOVA ^***^p<0.001 with ANOVA. A.U., arbitrary units.

**Figure 4:**
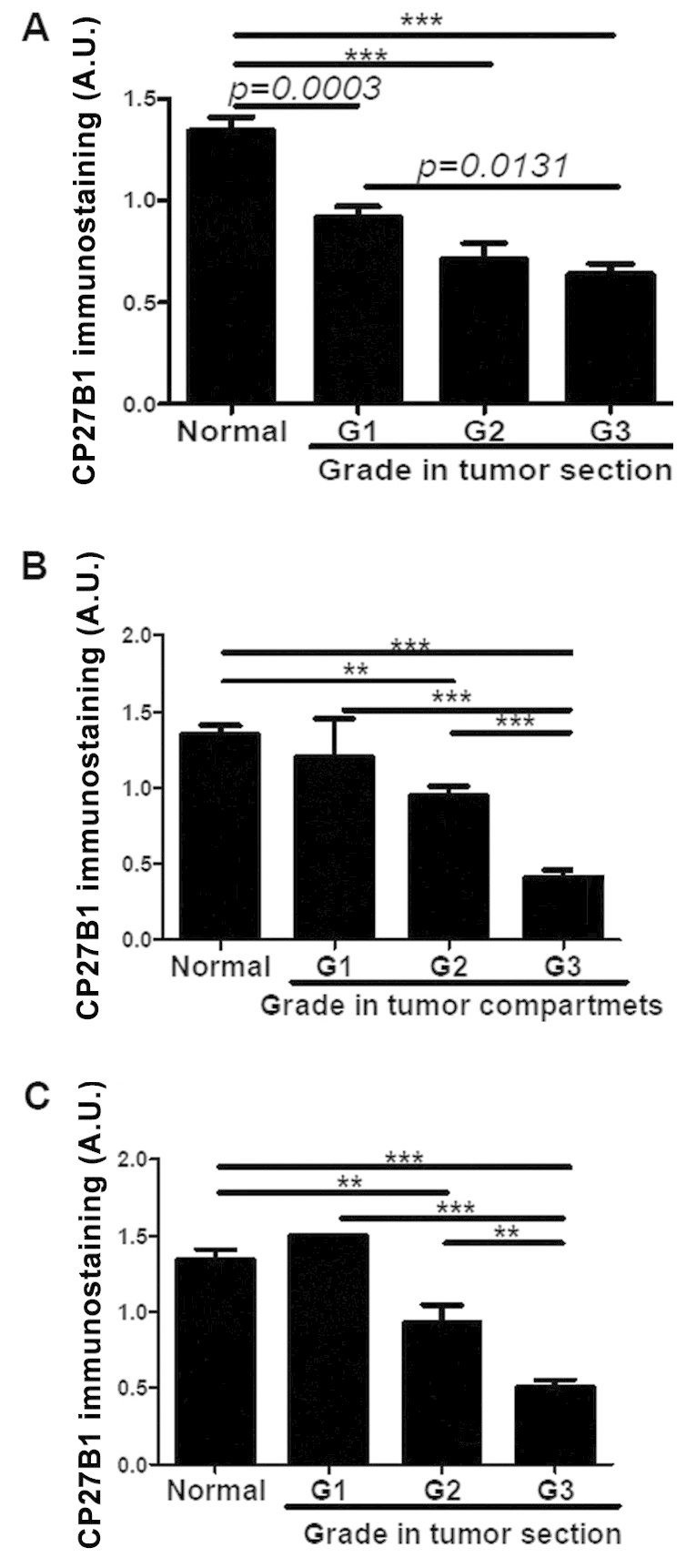
CYP27B1 immunostaining is inversely associated with tumor grade in primary cancers stratified according to tumor grade in whole section (A), tumor grade in separate compartment of the tumor (B) and in metastases (C). Statistically significant differences are denoted with p-values as determined by Student’s t-test and with asterisks by ANOVA ^**^p<0.01 and ^***^p<0.001. A.U., arbitrary units.

In the primary tumors and metastases, CYP27B1 was significantly decreased when compared to the normal epithelium (Figs. 2 and 3). In tumors classified according to grade in whole cancer material, there were statistically significant differences in CYP27B1 levels only between G1 and G3 tumors. However, in cancers classified according to tumor grade in the analyzed section, a gradual decrease in CYP27B1 was observed with increasing tumor grade, and a strong negative correlation with CYP27B1 immunostaining was found (r=−0.4206, p=0.0002). A similar trend was observed for CYP27B1 immunostaining and the grade of tumor compartments in metastases (r=−0.6009, p<0.0001). The CYP27B1 level in G1 was significantly higher than the level in G2 or G3 tumors. Comparable statistically significant trends and differences were observed for metastases (r=−0.4809, p=0.0217 and r=−0.6878, p<0.0001 for grades in sections and tumor compartments, respectively; Fig. 4A–C).

There were no relationships between CYP27B1 expression and the histological type of ovarian cancer and the presence of solid compartments in the tumor (data not shown).

### CYP27B1 and proliferative activity

**Figure 5:**
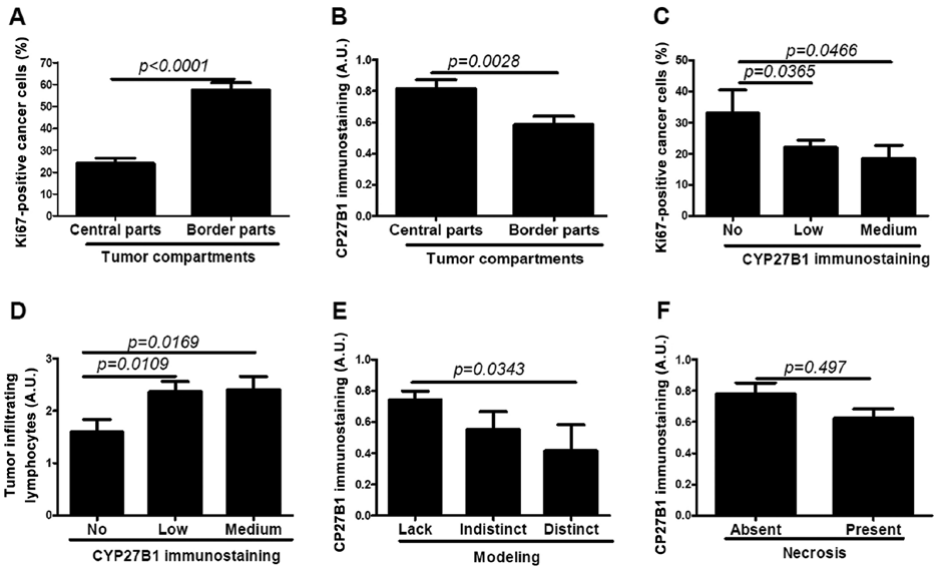
(A) Proliferation activity in the border and central parts of cancers. CYP27B1 immunostaining is associated with dynamism of tumor growth (B), Ki67 expression (C), stromal modeling pattern (D), lymphocyte infiltration (E) and necrosis (F). Statistically significant differences are denoted with p-values as determined by Student’s t-test. A.U., arbitrary units.

New parts of the tumors (borders) showing newly invading cancer nests demonstrated a significantly increased number of Ki67-positive cells when compared with the central, older parts of the cancer, without the presence of small groups of tumor cells or stromal modeling pattern (Fig. 5A). Likewise, CYP27B1 was differentially expressed within borders and central parts of the cancer with significantly stronger CYP27B1 expression in older tumor compartments (Fig. 5B). Subsequent analysis revealed statistically significant differences in the percentage of Ki67-positive cancer cells between regions without CYP27B1 expression and compartments with low and medium CYP27B1 expression (Fig. 5C). This association was more pronounced for central, more differentiated parts of the tumors with lower proliferative activity. However, we did not find such a relationship for rapidly proliferating border parts of the tumors (data not shown).

### CYP271 and the tumor microenvironment

CYP27B1 staining in tumor cells was positively correlated with TILs (r=0.2356, p=0.489). The ovarian cancers showing CYP27B1 expression demonstrated significantly higher lymphocytic infiltration (Fig. 5D). The CYP27B1 immunostaining was also negatively correlated with a stromal modeling pattern, and tumors with distinct modeling showed significantly lower CYP27B1 expression (r=−0.2643, p=0.0462, Fig. 5E). Furthermore, we found that in tumors with necrosis, CYP27B1 immunostaining intensity was significantly lower (Fig. 5F).

### CYP27B1 and ovarian cancer patient survival

**Figure 6:**
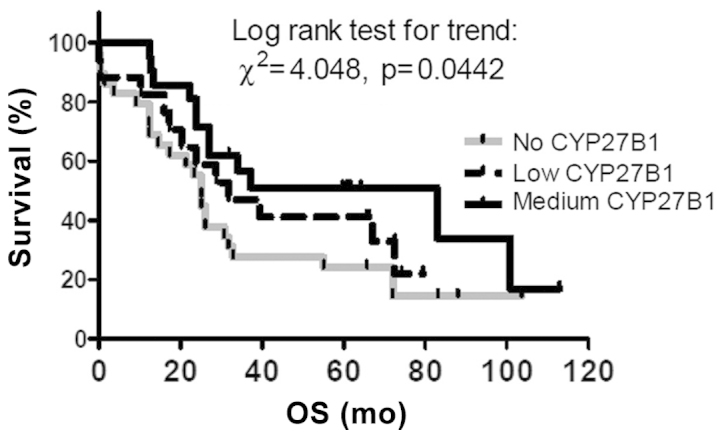
Lack of CYP27B1 in ovarian cancers is associated with shorter OS. OS, overall survival.

Analysis of the OS time revealed that CYP27B1 expression was associated with longer OS (median survival of patients with CYP27B1 and patients without CYP27B1 was 37.2 and 25 months, respectively). Kaplan-Meier survival curves also showed significant differences (log-rank test for trend χ^2^=4.048, p=0.044). These differences were more significant between cases without CYP27B1 and with medium CYP27B1 [log-rank (Mantel-Cox) test χ^2^ =4.270, p=0.039; log-rank test for trend χ^2^=5.559, p=0.018; median OS time: 25 vs. 82.80, respectively) (Fig. 6).

A similar trend was observed for disease-free survival; however, due to the low number of cases without metastases (n=6), these differences were not statistically significant (data not shown).

## Discussion

Since we previously found a correlation between expression of CYP27B1 and tumor behavior and clinical outcome in skin melanomas (48), we analyzed the expression of CYP27B1 in ovarian cancers. The expression of CYP27B1 was observed in 80.3% of primary ovarian cancers and in 94.6% of metastases. The CYP27B1 level was decreased in pathologic tissues in comparison to normal epithelium and was inversely correlated with tumor grade. Moreover, within tumor compartments with higher proliferative activity and newly invading cancer cells, CYP27B1 expression was clearly decreased. In addition, decreased CYP27B1 expression was accompanied by shorter OS. To the best of our knowledge this is the first report analyzing the *in situ* CYP27B1 protein expression in ovarian cancers, its correlation to prognostic factors and survival of ovarian cancer patients.

CYP27B1, activating 25(OH)D_3_ and elevating calcitriol levels, affects the biology of neighboring cells (8,53). Therefore, it is likely that disturbances in CYP27B1 expression can affect tumor development and progression. Reduced CYP27B1 expression has been observed in several tumors. Our previous research showed slightly elevated CYP27B1 levels in nevi and significantly reduced CYP27B1 in more advanced melanomas, lymph node metastases and melanoma cases that developed metastases. CYP27B1 levels were also negatively correlated with Ki67 expression, and a decrease in CYP27B1 was associated with significantly shorter OS (48). Similar associations have been found in other tumors, such as colon and breast cancers (24,37,54). In thyroid tumors, deregulation of vitamin D_3_ in the endocrine system was observed (55). It was characterized by the increased levels of VDR, CYP24A1 and CYP27B1 in benign and differentiated malignant thyroid tumors. However, the increase in CYP27B1 was not statistically significant. In addition, CYP27B1 was decreased in pN1 when compared to pN0 cases. Likewise, Hsu *et al* (56) observed a significant reduction in the CYP27B1 levels in benign and cancerous primary cell cultures and established prostate cancer cell lines when compared to normal prostate cells.

There is a shortage of information regarding CYP27B1 in ovarian cancers. Most of the published data concerned mRNA CYP27B1, and elevated expression of this gene was observed (57,58). Agic *et al* (58) also observed analogous differences in CYP27B1 at the protein level. A similar relationship with reference to CYP27B1 mRNA levels was found in ovarian tumor cell lines (59). In the present study, elevated levels of CYP27B1 mRNA were found but without significant differences at the protein level between benign and malignant cell lines. Although no differences were found between mRNA levels in the benign and malignant tissues, the protein level was significantly lower in the cancer tissues as in our studies. In the present study, we observed higher Ki67 expression within the regions that lacked CYP27B1, suggesting its contribution to the antiproliferative effect. Similarly, in thyroid cancers, the high Ki67 expression was accompanied by a loss of CYP27B1 (55).

An upregulation of CYP27B1 mRNA reported in several studies on breast, renal cancers or squamous cell carcinoma (57,60,61) requires an explanation. Here, it should be emphasized that protein levels can differ from mRNA levels due to the alternative splicing of CYP27B1 leading to production of several splice variants (59,62,63). The expression of CYP27B1 splice variants can be cell- and tissue-specific (62) and affect the levels of active enzyme or the inactive splice variant could not be translated, resulting in a reduction in protein synthesis. Several splice variants have been identified in ovarian cell lines and ovarian benign and malignant tissues, while no splice variants have been found in human benign granulosa cells (59,64).

Tumor-infiltrating lymphocytes (TILs) exhibit prognostic value in various malignant tumors, including ovarian cancers (51,52). Vitamin D_3_ exhibits immunoregulatory functions, and it regulates both normal innate and adaptive immunity (5). In research on patients with head and neck squamous cell cancers, treatment with 1,25(OH)_2_D_3_ resulted in increased intratumoral levels of CD4^+^ and CD8^+^ cells (10). In the present study, we found higher levels of TILs in tumors with higher CYP27B1. This may suggest that local activation of vitamin D_3_ by CYP27B1 can influence an immune response against cancer cells.

We observed reduced OS in patients when CYP27B1 was absent. Likewise, in our previous study, the absence of CYP27B1 protein in cutaneous melanomas was associated with shorter overall and disease-free survival (48). Our results are also in accordance with the analysis of the influence of systemic 25(OH)D_3_ level on survival of ovarian cancer patients showing reduced OS in the subgroup with severe deficiency of calcitriol (45). These results clearly indicate that both systemic and local vitamin D_3_ homeostasis influence ovarian cancer biology. In summary, we demonstrated a decrease in CYP27B1 expression in ovarian cancers. Our results indicate that local activation of vitamin D_3_ by the CYP27B1 enzyme in ovarian cancers affects tumor biology, and lack of CYP27B1 appears to be associated with a more aggressive phenotype of the tumor. These results also support the tumorostatic role of calcitriol in ovarian cancer biology.

## Figures and Tables

**Table I:** Pathomorphological characteristics of the ovarian cancer patients included in the present study.

Features	No. of cases
Age (years)	
Mean	56.9
Range	(25.6–81.0)
<40	1
41–50	10
51–60	31
61–70	12
>70	7
Grade	
G1	6
G2	23
G3	32
Histological type	
Borderline	3
Serous adenocarcinoma	43
Clear-cell carcinoma	5
Endometrioid cancer	8
Mucinous cancer	2
Necrosis	
Absent	25
Present	36
Stromal modeling pattern	
Absent	42
Present	19
Metastases	
Absent	15
Present	46
